# Factors associated with prolonged on-scene time in ambulance transportation among patients with minor diseases or injuries in Japan: a population-based observational study

**DOI:** 10.1186/s12873-023-00927-2

**Published:** 2024-01-07

**Authors:** Keiko Ueno, Chie Teramoto, Daisuke Nishioka, Shiho Kino, Hiroyuki Sawatari, Kazuaki Tanabe

**Affiliations:** 1 Department of Social Epidemiology, Graduate School of Medicine and School of Public Health, Kyoto University, Floor 2, Science Frontier Laboratory, Yoshidakonoe-cho, Sakyo-ku, Kyoto-shi, 606-8315 Kyoto, Japan; 2 Department of Perioperative and Critical Care Management, Graduate School of Biomedical and Health Sciences, Hiroshima University, Hiroshima, Japan; 3 Department of Medical Statistics, Research & Development Center, Osaka Medical and Pharmaceutical University, Osaka, Japan; 4 Department of Oral Health Promotion, Graduate School of Medical and Dental Sciences, Tokyo Medical and Dental University, Tokyo, Japan

**Keywords:** Ambulance, COVID-19 pandemic, Emergency medical services, Observational study, Prehospital emergency care

## Abstract

**Background:**

Prolonged prehospital time is a major global problem in the emergency medical system (EMS). Although factors related to prolonged on-scene times (OSTs) have been reported in patients with trauma and critical medical conditions, those in patients with minor diseases or injuries remain unclear. We examined factors associated with prolonged OSTs in patients with minor diseases or injuries.

**Methods:**

This population-based observational study used the ambulance transportation and request call record databases of the Higashihiroshima Fire Department, Japan, between January 1, 2016, and December 31, 2022. The participants were patients with minor diseases or injuries during the study period. We performed a multivariable logistic regression analysis with robust error variance to examine the association between patient age, sex, severity, accident type, date and time of ambulance call, and the coronavirus disease 2019 (COVID-19) pandemic with prolonged OSTs. Prolonged OST was defined as ≥ 30 min from the ambulance arrival at the scene to departure.

**Results:**

Of the 60,309 people transported by ambulance during the study period, 20,069 with minor diseases or injuries were included in the analysis. A total of 1,241 patients (6.2%) experienced prolonged OSTs. Fire accidents (adjusted odds ratio [aOR]: 7.77, 95% confidence interval [CI]: 3.82–15.79), natural disasters (aOR: 28.52, 95% CI: 2.09–389.76), motor vehicle accidents (aOR: 1.63, 95% CI: 1.30–2.06), assaults (aOR: 2.91, 95% CI: 1.86–4.53), self-injuries (aOR: 5.60, 95% CI: 3.37–9.32), number of hospital inquiries ≥ 4 (aOR: 77.34, 95% CI: 53.55–111.69), and the COVID-19 pandemic (aOR: 2.01, 95% CI: 1.62–2.50) were associated with prolonged OSTs. Moreover, older and female patients had prolonged OSTs (aOR: 1.18, 95% CI: 1.01–1.36 and aOR: 1.12, 95% CI: 1.08–1.18, respectively).

**Conclusions:**

Older age, female sex, fire accidents, natural disasters, motor vehicle accidents, assaults, self-injuries, number of hospital inquiries ≥ 4, and the COVID-19 pandemic influenced prolonged OSTs among patients with minor diseases or injuries. To improve community EMS, we should reconsider how to intervene with potentially modifiable factors, such as EMS personnel performance, the impact of the presence of allied services, hospital patient acceptance systems, and cooperation between general emergency and psychiatric hospitals.

**Supplementary Information:**

The online version contains supplementary material available at 10.1186/s12873-023-00927-2.

## Background

Prolonged prehospital time is a major global problem in emergency medical systems (EMSs). Prehospital times are divided into three parts: (1) response time, the time from the initial ambulance call to ambulance arrival at the scene; (2) on-scene time (OST), the time from ambulance arrival at the scene to departure; and (3) transport time, the time from ambulance departure at the scene to arrival at the hospital [1]. Response time is influenced by the number of available EMS units, the setup of the ambulance station, and the distance between the ambulance station and the scene. OST is affected by various activities at the scene, such as interviewing all related individuals, calling hospitals to accept the patients, performing detailed observations, and conducting prehospital procedures [2]. The transport time is mainly determined by the distance between the scene and the hospital. Because the response and transport times are relatively fixed, the OST is the time when we can intervene to minimize and improve EMS delays. Moreover, OSTs were found to be the longest, causing a total EMS delay [1, 3]. Prolonged OSTs are associated with poor neurologic outcomes in patients with cardiac arrest [4, 5] and increased in-hospital mortality in patients with trauma [1, 6–10]. In Japan, prolonged OSTs have become prominent, particularly during the coronavirus disease 2019 (COVID-19) pandemic [11].

The Japanese EMS is managed by local governments. People who need ambulance transportation to hospitals can request emergency services by dialing “119,” which directly links them to the dispatch center situated in the fire defense headquarters of the local government. Each ambulance has three EMS personnel; at least one is a nationally certified Emergency Life-Saving Technician. After arriving at the scene, EMS personnel evaluate the patient and provide emergency care and treatment, if necessary. Emergency Life-Saving Technicians can perform lifesaving procedures according to local protocols under the direction of physicians for cardiac arrest, critical shock, or unconsciousness due to hypoglycemia. These procedures include securing the airway, establishing intravenous access, administering epinephrine, and measuring blood glucose levels after IV glucose administration [12]. Then, while remaining at the scene, they select the most suitable hospital for the patients based on the severity of their illness/injury and make request calls to hospitals until they accept the patients. Once the nearby emergency hospital agrees to treat the patient, the patient is transported by ambulance. All patients, except those who refuse to go to the hospital or die, are transported to the hospital. The cost of care for EMS personnel and transportation is covered by local governments, and there is no charge for ambulance users [12].

Previous studies have reported the following factors related to prolonged OSTs: older age, female sex, blunt trauma, nighttime, and holidays in patients with trauma [7, 13, 14], and older age, female sex, minor diseases, intoxication, winter season, hospital type, and geographical areas in those with critical medical conditions [15, 16]. However, factors related to prolonged OSTs in patients with minor diseases or injuries remain unclear. Globally, the number of ambulance calls for minor diseases or injuries continues to increase [17]. In Japan, ambulance users with minor diseases or injuries, defined according to the national criteria as those with minor injuries or diseases who do not require hospitalization and are discharged from the emergency department (ED), account for approximately 45% of all ambulance users [18]. Thus, we aimed to examine the factors associated with prolonged OSTs in ambulance transportation among patients with minor diseases or injuries.

## Methods

### Study design and participants

This population-based observational study was conducted in three cities under the jurisdiction of the Higashihiroshima Fire Department in Hiroshima, Japan. Patients with minor diseases or injuries between January 1, 2016, and December 31, 2022, were included in this study. In Japan, the severity of an ambulance user’s condition is based on the physician’s judgment at the time of the initial ED visit and is defined in the national criteria of emergency statistics as follows: a mild condition is defined as an injury or disease that does not require hospitalization and treatment, a moderate condition is defined as an injury or disease that requires hospitalization and treatment for less than three weeks, a severe condition is defined as an injury or disease that requires hospitalization and treatment for three weeks or more, and death. Thus, patients with minor diseases or injuries did not require hospitalization and were discharged from the ED [18]. The study protocol was approved by the Ethics Committee of the Graduate School and Faculty of Medicine of Kyoto University (approval no. R3745). The Ethics Committee of the Graduate School and the Faculty of Medicine of Kyoto University allowed us to perform the study without informed consent because of the anonymity of the data collected for routine operations and the retrospective nature of the study.

### Setting

**Fig. 1:**
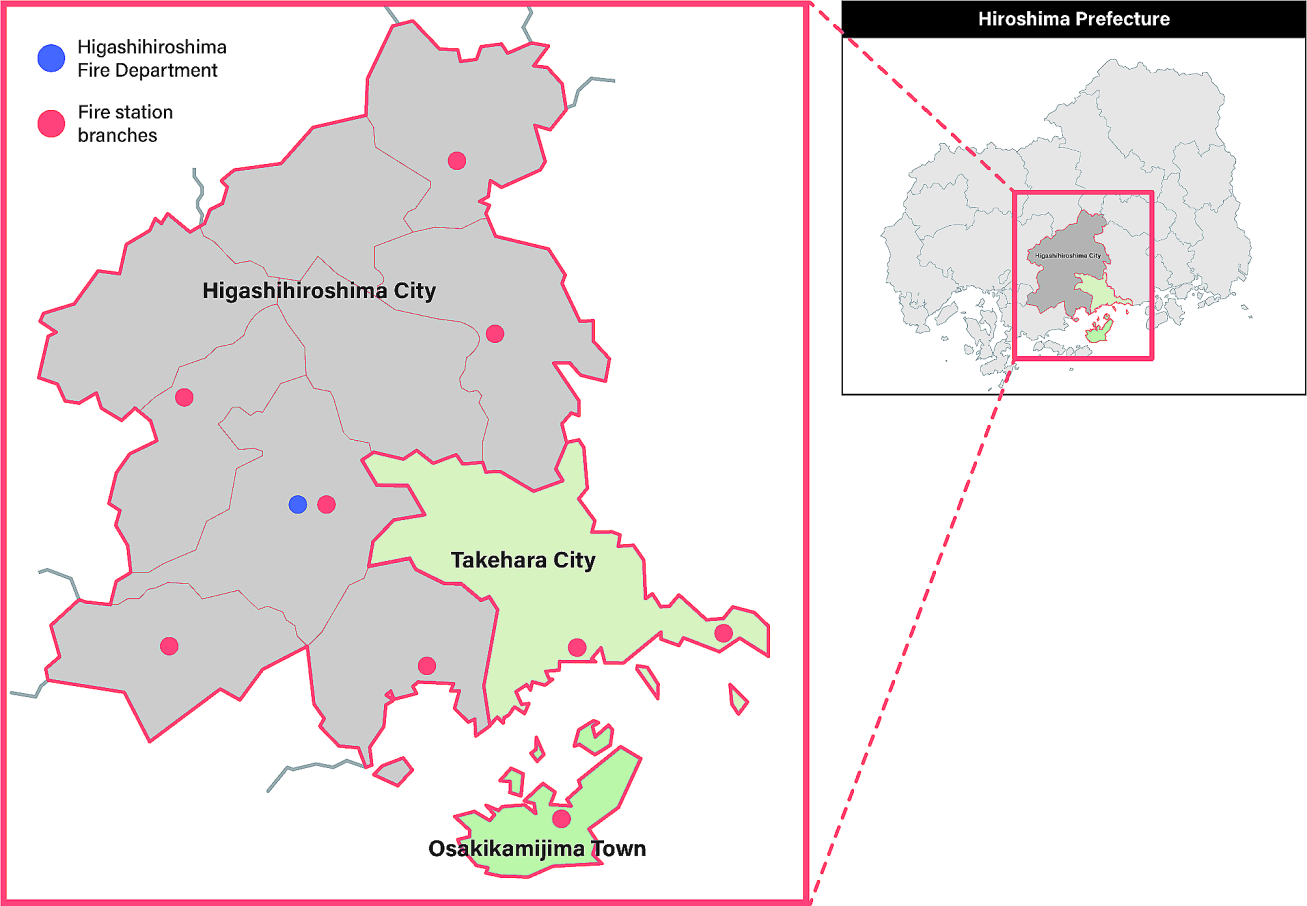
Location and map of three local cities under the jurisdiction of the Higashihiroshima Fire Department (created by the authors)

The Higashihiroshima Fire Department is responsible for ambulance services in Higashihiroshima City, Takehara City, and Osakikamijima Town. The locations and maps of these cities are shown in Fig. 1. In 2022, Higashihiroshima City had a population of 189,039 individuals with a population density of 298 persons per square kilometer. Takehara City had a population of 24,071, with a population density of 204 persons per square kilometer. Osakikamijima Town is an island located in the center of the Seto Inland Sea, with a population of 7,153 and a population density of 166 persons per square kilometer. In 2022, there were nine and three secondary emergency centers in Higashihiroshima City and Takehara City, respectively. There were no secondary emergency centers in Osakikamijima Town or tertiary emergency centers in these three cities. Patients in Osakikamijima Town were transferred by emergency boats or helicopters to secondary and tertiary emergency centers on the main island when necessary. EMS personnel recorded the initial medical data of all patients transported to the hospital and asked the physicians in charge at the ED to document their severities on the recording paper. The Higashihiroshima Fire Department retained these data.

### Data sources

The data were obtained with permission from the Higashihiroshima Fire Department. The data sources were the ambulance transportation and ambulance request call record databases from the Higashihiroshima Fire Department. Databases were merged using personal identification numbers. The database included information on the age and sex of the patient, date and time of the ambulance call, accident category, prehospital time including OST, number of phone calls to hospitals from EMS personnel, and the fire station from which the ambulance was dispatched.

### Measurements

#### Outcome variable

The primary outcome of this study was prolonged OSTs. In Japan, prolonged OSTs are defined as ≥ 30 min from ambulance arrival at the scene to departure according to the guidelines of the Fire and Disaster Management Agency of the Ministry of Internal Affairs and Communications [19]. We dichotomized prolonged OSTs into binary variables that identified OSTs ≥ 30 min or < 30 min.

#### Explanatory variables

The following information was extracted as explanatory variables: age, sex, severity, accident type, date and time of ambulance call, number of hospital inquiries, and year and month of ambulance transport. Age and accident type were categorized according to the Fire and Disaster Management Agency classification [18]. Age was divided into 5 groups: newborns (< 28 days), infants (28 days to 6 years), adolescents (7–17 years), adults (18–64 years), and older adults ( ≥ 65 years). The accident types were divided into 12 groups: fire accidents (accidents directly resulting from a fire at the scene, including major and minor fires), natural disasters (accidents caused by storms, torrential rains, heavy snowfalls, floods, storm surges, earthquakes, tsunamis, eruptions, avalanches, landslides, and other disasters resulting from unusual natural phenomena), water-related accidents (accidents caused by drowning or falling into the water while swimming), motor vehicle accidents (accidents caused by collisions and contact between all traffic, by a single accident, or by pedestrians, etc. coming into contact with traffic), work-related accidents (accidents that occur while working in factories, business sites, workshops, construction sites, etc.), sports-related accidents (accidents that occur while participating in athletic events, including those involving athletes, referees, and related personnel), other types of accidents (unexpected accidents that are not classified as any other types), assaults (accidents in which one person intentionally inflicts damage on another person), self-injuries (accidents in which one person intentionally inflicts injury on themselves), acute illnesses, interhospital transport (transport of patients from one medical facility to another), and others (those which cannot be classified into the above types) [20]. Interhospital transport patients were excluded from the analysis. The dates and times of the ambulance calls were divided into 6 groups: weekday daytime (9:00–16:00), weekday early night (17:00–0:00), weekday late night (1:00–8:00), weekend daytime (9:00–16:00), weekend early night (17:00–0:00), and weekend late night (1:00–8:00). The Fire and Disaster Management Agency of the Ministry of Internal Affairs and Communications defined ambulance transport with ≥ 4 phone calls to hospitals until patients are accepted as “difficult-to-transfer cases” [19]. We dichotomized the number of hospital inquiries into binary variables exhibited more than four times. The COVID-19 pandemic was classified as “pre-pandemic” from January 2016 to March 2020 and “pandemic” from April 2020 to December 2022 based on the number of infected patients in the participating cities [21].

### Statistical analyses

First, we described the characteristics of all the study participants in relation to OSTs during the study period. Second, we performed univariable logistic regression analysis and calculated the crude odds ratios (ORs) of prolonged OSTs and the 95% confidence interval (CI) for each explanatory variable. Third, we performed a multivariable logistic regression analysis with robust error variance to calculate the adjusted OR (aOR) of each explanatory variable to investigate the factors related to prolonged OSTs. To adjust for possible geographical variations, the fire stations from which the ambulances were dispatched were included as dummy variables. These were divided into nine geographical areas. This enabled us to adjust for unobserved regional variations in the EMSs. Furthermore, we performed an additional analysis that divided participants into two groups: those who used an ambulance during the pandemic period (Analysis S1) and those who used an ambulance during the pre-pandemic period (Analysis S2), since the COVID-19 pandemic is a known factor associated with prolonged OSTs [11, 22–29]. Lastly, we performed an additional analysis that excluded participants transported by ambulance from the Osakikamijima Fire Station (Analysis S3) because Osakikamijima Town had a unique condition: patients were transferred by emergency boats or helicopters to secondary and tertiary emergency centers on the main island when necessary. Statistical analyses were conducted using STATA SE (version 17.0; Stata Corp., College Station, Texas, USA). This study was conducted in accordance with the STROBE (Strengthening the Reporting of Observational Studies in Epidemiology) recommendations.

## Results

**Fig. 2:**
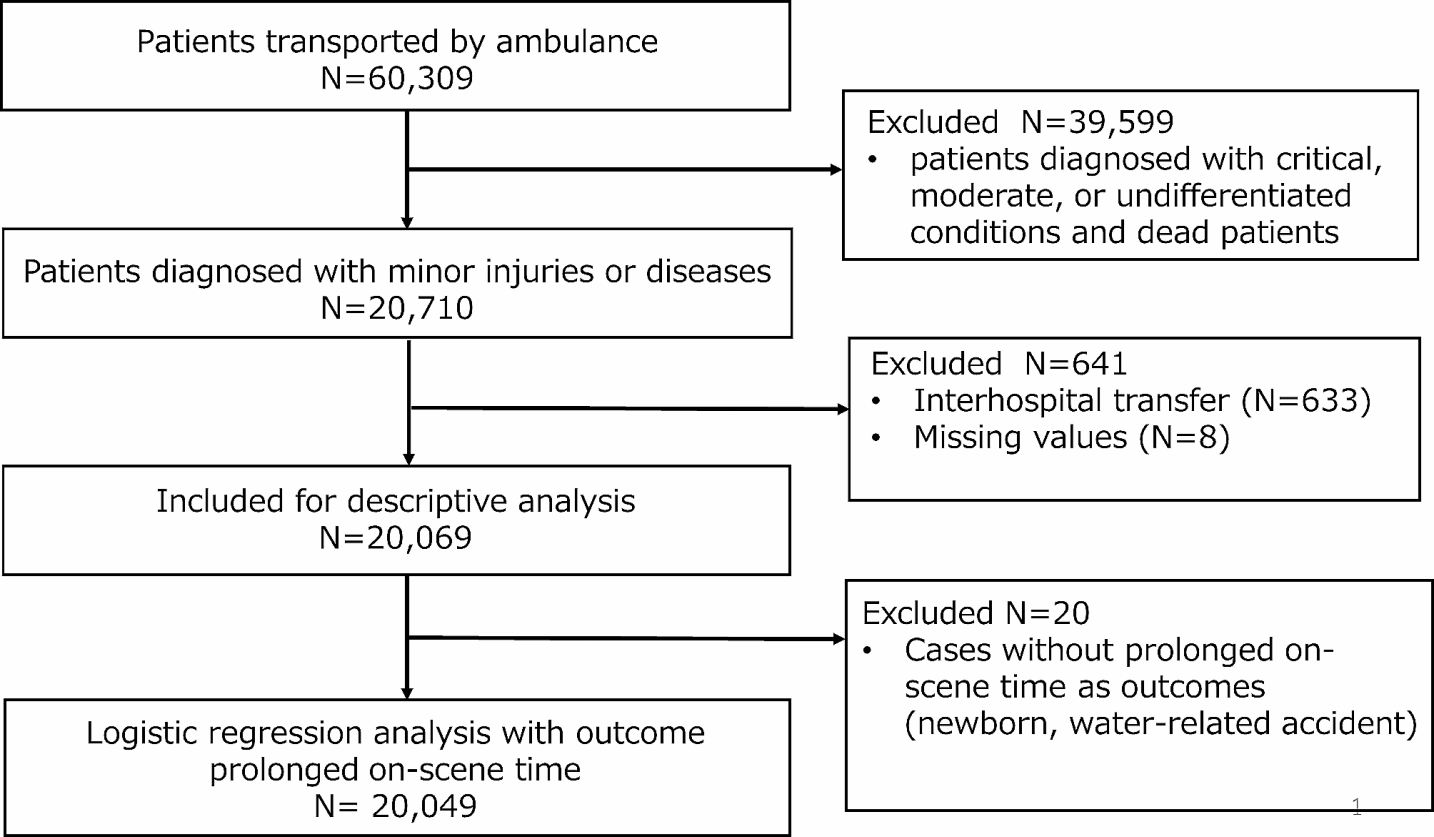
Flowchart of study participants

Of the 60,309 patients transported by ambulance during the study period, the patients with minor diseases or injuries were 20,710. Among them, we included 20,069 patients in the analysis (Fig. 2). Most patients were adults aged 18–64 years (43.1%) or older (43.2%). A total of 62.5%, 17.4%, and 17.1% of the patients were categorized as having acute illnesses, other types of injuries, and motor vehicle accidents, respectively. A total of 1,241 patients (6.2%) experienced prolonged OSTs (Table 1). Among patients with prolonged OSTs, the mean OST was 40.5 min (standard deviation:16.1 min; minimum 30 min; maximum 272 min).

**Table 1:** Baseline characteristics of the study participants

	Total participants N (%) (N = 20,069)	Patients with prolonged OST (n = 1,241, 6.2%) n (% for N)
Age		
Newborns	17(0.1)	0, 0.0%
Infants	1,627 (8.1)	53, 3.3%
Adolescents	1,109 (5.5)	55, 5.0%
Adults	8,642 (43.1)	575, 6.7%
Older people	8,674 (43.2)	558, 6.4%
Sex		
Male	10,798 (53.8)	652, 6.0%
Female	9,271 (46.2)	589, 6.4%
Accident type		
Acute illnesses	12,544 (62.5)	717, 5.7%
Fire accidents	57 (0.3)	15, 26.3%
Natural disasters	14 (0.1)	7, 50.0%
Water-related accidents	3 (0.0)	0, 0.0%
Motor vehicle accidents	3,432 (17.1)	266, 7.6%
Work-related accidents	188 (0.9)	12, 6.4%
Sports-related accidents	149 (0.7)	2, 1.3%
Other types of accidents	3,499 (17.4)	184, 5.3%
Assaults	91 (0.5)	13, 14.3%
Self-injuries	85 (0.4)	24, 28.2%
Others	7 (0.0)	1, 14.2%
Dates and times of the ambulance calls		
Weekday daytime (9–16)	5,567 (27.7)	372, 6.7%
Weekday early night (17–0)	4,943 (24.6)	329, 6.7%
Weekday late night (1–8)	3,284 (16.4)	210, 6.4%
Weekend daytime (9–16)	2,680 (13.4)	133, 5.0%
Weekend early night (17–0)	2,346 (11.7)	112, 4.9%
Weekend late night (1–8)	1,249 (6.2)	85, 6.8%
Number of hospital inquiries		
< 4	19,858 (98.9)	1.073, 5.4%
≥ 4	211 (1.1)	168, 79.6%
COVID-19 pandemic period		
Pre-pandemic period (1/2016–3/2020)	12,721 (63.4)	604, 4.7%
Pandemic period (4/2020–12/2022)	7,348 (36.6)	637, 8.7%

OST, on-scene time.; COVID-19, Coronavirus disease 2019

The results of univariable and multivariable logistic regression analyses for prolonged OSTs are shown in Table 2. Univariable logistic regression analysis showed that fire accidents, natural disasters, motor vehicle accidents, assaults, self-injuries, number of hospital inquiries ≥ 4, and the COVID-19 pandemic were associated with prolonged OSTs. In the multivariable logistic regression analysis, fire accidents (aOR: 7.77, 95% CI: 3.83–15.79), natural disasters (aOR: 28.52, 95% CI: 2.09–389.76), motor vehicle accidents (aOR: 1.63, 95% CI: 1.30–2.06), assaults (aOR: 2.91, 95% CI: 1.86–4.53), self-injuries (aOR: 5.60, 95% CI: 3.37–9.32), number of hospital inquiries ≥ 4 (aOR: 77.34, 95% CI: 53.55–111.69), and the COVID-19 pandemic (aOR: 2.01, 95% CI: 1.62–2.50) continued to be associated with prolonged OSTs. Additionally, older and female patients had longer OSTs (aOR: 1.18, 95% CI: 1.01–1.36 and aOR; 1.12, 95% CI: 1.08–1.18, respectively). The dates and times of ambulance calls were not associated with prolonged OSTs.

**Table 2:** Odds ratios (ORs) and 95% confidence intervals (CIs) for prolonged on-scene time (OST): Results of univariable and multivariable logistic regression analyses

	Crude			Adjusted*		
	OR	95% CI		OR	95% CI	
Age						
Infants	0.47	0.35	0.63	0.54	0.40	0.71
Adolescents	0.73	0.55	0.97	0.79	0.53	1.27
Adults	Ref			Ref		
Older people	0.96	0.86	1.09	1.18	1.01	1.36
Sex						
Male	Ref			Ref		
Female	1.06	0.94	1.18	1.12	1.08	1.18
Accident type						
Acute illnesses	Ref			Ref		
Fire accidents	5.89	3.25	10.67	7.77	3.83	15.79
Natural disasters	16.50	5.77	47.15	28.52	2.09	389.76
Motor vehicle accidents	1.39	1.20	1.60	1.63	1.30	2.06
Work-related accidents	1.12	0.62	2.03	1.51	0.78	2.96
Sports-related accidents	0.22	0.06	0.91	0.20	0.04	0.94
Other types of accidents	0.92	0.78	1.08	0.92	0.79	1.07
Assaults	2.74	1.52	4.97	2.91	1.86	4.53
Self-injuries	6.49	4.02	10.47	5.60	3.37	9.32
Others	2.75	0.33	22.87	3.17	0.26	38.71
Dates and times of the ambulance calls						
Weekday daytime (9–16)	Ref			Ref		
Weekday early night (17–0)	1.00	0.85	1.16	1.01	0.82	1.25
Weekday late night (1–8)	0.95	0.80	1.14	0.96	0.63	1.47
Weekend daytime (9–16)	0.73	0.59	0.89	0.71	0.44	1.14
Weekend early night (17–0)	0.70	0.56	0.87	0.68	0.54	0.85
Weekend late night (1–8)	1.02	0.80	1.30	1.00	0.79	1.27
Number of hospital inquiries						
< 4	Ref			Ref		
≥ 4	68.40	48.66	96.15	77.34	53.55	111.69
COVID-19 pandemic period						
Pre-pandemic period (1/2016–-3/2020)	Ref			Ref		
Pandemic period (4/2020–-12/2020)	1.90	1.70	2.14	2.01	1.62	2.50

*To adjust for possible geographical variations, the fire stations from which the ambulances were dispatched were included as dummy variables.

The categories of newborns and water-related accidents were not shown because they did not have an outcome of prolonged OST.

COVID-19, Coronavirus disease 2019; OST, on-scene time; OR, odds ratio; CI, confidence interval

Our additional analysis (Analysis S1), which included only patients with minor diseases or injuries who used ambulances during the pandemic period, showed similar results to those of the main analysis (Table S1 in Additional file 1). The results of Analysis S2 showed that fire accidents, natural disasters, motor vehicle accidents, assaults, self-injuries, and number of hospital inquiries ≥4 were factors associated with prolonged OSTs among patients with minor diseases or injuries who used an ambulance during the pre-pandemic period (Table S2 in Additional file 2). Moreover, the results of Analysis S3 were the same as those of the main analysis (Tables S3 and S4 in Additional File 3).

## Discussion

The present study found that older patients, females, fire accidents, natural disasters, motor vehicle accidents, assaults, self-injuries, number of hospital inquiries ≥ 4, and the COVID-19 pandemic were factors associated with prolonged OSTs in ambulance transportation among patients with minor diseases or injuries in local Japanese cities. To the best of our knowledge, this is the first study to focus on the factors associated with prolonged OSTs in patients with minor diseases or injuries.

The finding that self-injuries and assaults were associated with prolonged OSTs aligns with previous studies. A population-based observational study showed that OSTs were significantly longer in the self-injury group than in other accident categories [30]. Also, the OSTs in psychiatric cases are longer than those in medical and trauma cases [31]. For self-injured or assaulted patients, it may take some time for EMS personnel to persuade or obtain consent for someone else to call the ambulance, check their vital signs, take their information, and communicate with the hospital until they accept these patients. This finding is also related to how self-injured or assaulted patients are treated by the Japanese EMS. Treatment for these patients is often provided in general emergency hospitals that have difficulty dealing with patients who may need psychiatric evaluation and psychiatric hospitals that have difficulty managing their physical problems. Recent research in Japan revealed that hospitals with general and psychiatric inpatient beds and high-level emergency care centers had shorter prehospital times (combined with on-scene and transfer times) [32]. Thus, it is essential to establish cooperation and coordination of care between general emergency and psychiatric hospitals to reduce OST in ambulance transportation for self-injured and assaulted patients.

Fire accidents, natural disasters, and motor vehicle accidents are known predictors of prolonged OSTs [1, 33]. Performing patient extrication, assessment, emergency care, and procedures by EMS personnel, such as spinal immobilization and intravenous access, are required on the scene before transportation, regardless of disease or injury severity. These activities can account for prolonged OSTs. Furthermore, the presence of allied services such as police officers and firefighters can influence the OSTs after these accidents [33]. The waiting time for their arrival and communication with them tends to increase OSTs.

The finding that the number of hospital inquiries ≥ 4 was significantly associated with prolonged OSTs is consistent with several Japanese studies [13, 16, 34–35]. Making request calls to hospitals until the patients are accepted is unique to the Japanese emergency system. In some Japanese hospitals, it is customary for information clerks or nurses, not emergency physicians, to receive calls from EMS personnel. Subsequently, they ask the physician to determine if they can examine and treat the patient. Thus, this system lengthens the communication time between EMS personnel and hospitals and necessitates EMS personnel to make multiple calls to hospitals until the physician in charge accepts the patient. In addition, a lack of education on communication skills for hospital contact among EMS personnel has been noted as the cause of multiple hospital inquiries [36].

Our study showed that the COVID-19 pandemic was associated with prolonged OSTs. This result is consistent with studies conducted in Japan [11, 22–25] and other countries [26–28]. During the COVID-19 pandemic, several factors might have affected OSTs. For instance, EMS personnel had to use personal protective equipment, including face masks, gowns with arm sleeve covers and shoe covers, gloves, and eye protectors [29, 37]. They had to obtain more detailed information about patients, such as their exposure history to COVID-19 [26]. Furthermore, they spent longer on the scene until they found hospitals because some hospitals hesitated to accept febrile patients or those with flu-like symptoms that mimicked those of COVID-19 [38].

Older age is associated with prolonged OSTs in patients with trauma [14], those with road traffic injuries, highly-urgent transported patients [15], and transported patients [39]. Older people are likely to have more serious injuries than younger adults; thus, they may require a thorough assessment and a longer time for on-scene stabilization [39]. Furthermore, because they tend to have more complex medical conditions, prolonged OSTs in older patients may reflect the time required for EMS personnel to ask for information from them or their families. The present study also revealed that the female sex was associated with prolonged OSTs. Females had longer median or average OSTs than males in urgent cases [15], trauma [7], and coronary artery syndrome [40–42]. However, the plausible reasons for the prolonged OST among female patients in these cases and those with minor diseases or injuries are unclear. Future studies are needed to understand these sex differences.

Our results have several important implications. Factors influencing OSTs are multifaceted and comprise modifiable and unmodifiable factors [39]. In this study, patient age and sex, accident type, and the COVID-19 pandemic are unmodifiable factors that are beyond our control and we cannot change. Thus, we must reconsider how to intervene with potentially modifiable factors, including EMS personnel performance, the influence of the presence of allied services, acceptance systems for patients at hospitals, and the cooperation and coordination of care between general emergency and psychiatric hospitals. Various measures should be considered to address these modifiable factors. Regarding EMS personnel performance, EMS personnel need to be informed to perform only ABC assessments (assessing a patient’s airway, breathing, and circulatory status) prior to accommodating patients in an ambulance and conduct detailed observations after accommodating them in an ambulance when they review all the vital signs and perform detailed observations at the scene [2]. Additionally, an educational program for EMS personnel may be needed to enhance communication skills for hospital contact. The presence of allied services (police officers and firefighters) can influence OSTs, although it is imperative that EMS personnel cooperate with them at the scene of disasters or accidents. When patients have minor or no obvious injuries, the police may spend more time at the scene of a motor vehicle accident, asking those involved about the circumstances of the accident. It is necessary to establish a system in which police officers talk to patients after being examined at the hospital. As for patient acceptance systems at hospitals, the information transmission process should be simplified by promoting a “hotline” (a telephone line that allows EMS personnel to directly talk to the physicians in charge of the ED) and by creating structured communication procedures between EMS personnel and hospital staff when EMS personnel make request calls to hospitals. Additionally, there has been increasing advocacy for implementing Information and Communication Technology to improve hospital patient acceptance systems in Japan [43]. Several local governments have already begun using digital information devices such as tablets and smartphones to share patient information between EMS personnel and hospitals [43, 44]. Currently, patient information is mainly obtained orally from patients, their family members, or people who call an ambulance. The method of obtaining patient information, its accuracy, and the time required for gathering it are major issues when these people cannot provide information to EMS personnel. Utilizing an ICT system enables EMS personnel to collect patient information more quickly and share the same information with hospitals. Regarding cooperation and coordination of care between general emergency and psychiatric hospitals, the Japanese Association for Emergency Psychiatry recommends the following: (1) creating a forum for healthcare providers to exchange opinions and solve problems on a daily basis; (2) establishing a system for healthcare providers to receive training and learning on physical and mental disorders commonly encountered in clinical practice; (3) preparing a communication system at both medical institutions; and (4) providing incentives and other measures by the municipal government to encourage the development of cooperation between general emergency and psychiatric hospitals [45]. These recommendations would help facilitate cooperation and coordination of care between general emergency and psychiatric hospitals and ultimately contribute to reducing OSTs.

Our study had several limitations. First, the results may not apply to urban cities or other countries because this study was conducted in local Japanese cities. Second, the severity of the patient’s condition may not have been accurately reflected. This is because EMS personnel usually obtain the initial severity assessment results from the ED physician after hospital arrival, and their condition may change after further examination and testing in the ED. Thus, it is possible that some of the study participants may have been misclassified, and further studies using the final results of their severity from the hospital record database are needed. Third, some results had relatively wide CIs owing to the small sample sizes of the categories. The results for these categories should be replicated using a larger sample size. Fourth, there is no clear evidence showing that the cutoff value for OSTs is 30 min in the guidelines of the Fire and Disaster Management Agency of the Ministry of Internal Affairs and Communications. They reported that the majority of OSTs for patients with severe medical conditions accounted for < 30 min (52% of them were < 15 min and 42.5% of them were between 15 and 30 min), and only 5.0% of them were ≥ 30 min, and they noted that prolonged OSTs were defined as ≥ 30 min from ambulance arrival at the scene to departure [46]. Future studies are warranted to verify the appropriate cut-off values for prolonged OSTs. Finally, there might have been unmeasured factors not included in the analysis because of data limitations. These factors include the ability of EMS personnel to manage a scene [33], extrication time [8], amount of time to secure the scene [8], and hospital capacity [32]. Future studies should address these limitations.

## Conclusions

The present study demonstrated that older age, female sex, fire accidents, natural disasters, motor vehicle accidents, assaults, self-injuries, number of hospital inquiries ≥ 4, and the COVID-19 pandemic were related to prolonged OSTs in patients with minor diseases or injuries. We must reconsider how to intervene with potentially modifiable factors, such as the performance of EMS personnel, the impact of the presence of allied services, hospital patient acceptance systems, and cooperation and coordination of care between general emergency and psychiatric hospitals to improve EMS in the community.

### Electronic supplementary material

Below is the link to the electronic supplementary material.

Supplementary Material 1

Supplementary Material 2

Supplementary Material 3

## Data Availability

The datasets generated and analyzed during the current study are not publicly available because of their usage under license for the current study but are available from the corresponding author upon reasonable request and with permission from the participating municipal fire department.

## Abbreviations

aOR: Adjusted odds ratio
CI: Confidence interval
COVID-19: Coronavirus disease 2019
ED: Emergency department
EMS: Emergency medical system
OR: Odds ratio
OST: On-scene time
