# Supplementary material for: Factors associated with prolonged on-scene time in ambulance transportation among patients with minor diseases or injuries in Japan: a population-based observational study
Source: BMC Emerg Med. 2024 Jan 7;24:10. doi: 10.1186/s12873-023-00927-2 (PMC10773094; doi:10.1186/s12873-023-00927-2)
Supplement: Supplementary file 1 — Supplementary Material 1 [file 12873_2023_927_MOESM1_ESM.docx]

**Additional file 1**

| Table S2. Odds ratios (ORs) and 95% confidence intervals (CIs) for prolonged on-scene time (OST) among patients who used an ambulance during the COVID-19 pandemic period^*^ (n=7,287): Results of univariable and multivariable logistic regression analyses (Analysis S1) | | | | | | |
| --- | --- | --- | --- | --- | --- | --- |
|  | Crude | | | Adjusted^†^ | | |
|  | OR | 95% CI | | OR | 95% CI | |
| Age |  | | |  | | |
| Infants | 0.56 | 0.39 | 0.81 | 0.66 | 0.50 | 0.88 |
| Adolescents | 0.84 | 0.56 | 1.25 | 0.94 | 0.54 | 1.64 |
| Adults | Ref | | | Ref | | |
| Older people | 1.08 | 0.91 | 1.28 | 1.31 | 1.10 | 1.57 |
| Sex |  | | |  | | |
| Male | Ref | | | Ref | | |
| Female | 1.14 | 0.97 | 1.35 | 1.18 | 1.08 | 1.30 |
| Accident type |  | | |  | | |
| Acute illnesses | Ref | | | Ref | | |
| Fire accidents | 4.07 | 1.07 | 15.39 | 4.64 | 1.80 | 11.95 |
| Motor vehicle accidents | 1.34 | 1.08 | 1.66 | 1.53 | 1.26 | 1.85 |
| Work-related accidents | 1.16 | 0.50 | 2.71 | 1.57 | 0.54 | 4.54 |
| Other types of accidents | 0.79 | 0.63 | 1.00 | 0.78 | 0.65 | 0.93 |
| Assaults | 4.22 | 1.75 | 10.16 | 4.54 | 1.44 | 14.27 |
| Self-injuries | 6.02 | 2.76 | 13.14 | 4.80 | 1.93 | 11.98 |
| Others | 10.84 | 0.68 | 173.70 | 12.31 | 0.69 | 218.79 |
| Dates and times of the ambulance calls | | | |  | | |
| Weekday daytime (9–16) | Ref | | | Ref | | |
| Weekday early night (17–0) | 0.86 | 0.69 | 1.07 | 0.87 | 0.71 | 1.07 |
| Weekday late night (1–8) | 0.85 | 0.67 | 1.08 | 0.84 | 0.48 | 1.47 |
| Weekend daytime (9–16) | 0.62 | 0.47 | 0.83 | 0.64 | 0.38 | 1.08 |
| Weekend early night (17–0) | 0.58 | 0.42 | 0.79 | 0.60 | 0.45 | 0.78 |
| Weekend late night (1–8) | 0.75 | 0.52 | 1.07 | 0.73 | 0.54 | 0.99 |
| Number of hospital inquiries | | | |  |  |  |
| <4 | Ref | | | Ref | | |
| ≥4 | 82.59 | 45.83 | 148.81 | 89.75 | 46.97 | 171.48 |
| *The pandemic period was between April 2020 and December 2022.  † To adjust for possible geographical variations, the fire stations from which the ambulances were dispatched were included as dummy variables.  The categories of newborns, natural disasters, water-related accidents, and sports-related accidents were not shown because they did not have an outcome of prolonged OST. | | | | | | |

COVID-19, Coronavirus disease 2019; OST, on-scene time; OR, odds ratio; CI, confidence interval
