# Supplementary material for: Factors associated with prolonged on-scene time in ambulance transportation among patients with minor diseases or injuries in Japan: a population-based observational study
Source: BMC Emerg Med. 2024 Jan 7;24:10. doi: 10.1186/s12873-023-00927-2 (PMC10773094; doi:10.1186/s12873-023-00927-2)
Supplement: Supplementary file 2 — Supplementary Material 2 [file 12873_2023_927_MOESM2_ESM.docx]

**Additional file 2**

| Table S1. Odds ratios (ORs) and 95% confidence intervals (CIs) for prolonged on-scene time (OST) among patients who used an ambulance during the COVID-19 pre-pandemic period^*^ (n=12,700): Results of univariable and multivariable logistic regression analyses (Analysis S2) | | | | | | |
| --- | --- | --- | --- | --- | --- | --- |
|  | Crude | | | Adjusted^†^ | | |
|  | OR | 95% CI | | OR | 95% CI | |
| Age |  | | |  | | |
| Infants | 0.36 | 0.23 | 0.57 | 0.41 | 0.26 | 0.64 |
| Adolescents | 0.65 | 0.43 | 0.98 | 0.65 | 0.39 | 1.11 |
| Adults | Ref | | | Ref | | |
| Older people | 0.87 | 0.73 | 1.03 | 1.06 | 0.82 | 1.38 |
| Sex |  | | |  | | |
| Male | Ref | | | Ref | | |
| Female | 1.00 | 0.85 | 1.18 | 1.08 | 0.96 | 1.22 |
| Accident type |  | | |  | | |
| Acute illnesses | Ref | | | Ref | | |
| Fire accidents | 8.32 | 4.27 | 16.23 | 9.71 | 4.25 | 22.20 |
| Natural disasters | 33.02 | 10.42 | 104.60 | 45.37 | 2.95 | 696.90 |
| Motor vehicle accidents | 1.58 | 1.29 | 1.93 | 1.74 | 1.28 | 2.36 |
| Work-related accidents | 1.18 | 0.52 | 2.70 | 1.46 | 0.56 | 3.82 |
| Sports-related accidents | 0.51 | 0.13 | 2.09 | 0.37 | 0.07 | 2.11 |
| Other types of accidents | 1.05 | 0.83 | 1.33 | 1.11 | 0.83 | 1.47 |
| Assaults | 2.36 | 1.01 | 5.50 | 2.12 | 1.34 | 3.35 |
| Self-injuries | 7.68 | 4.16 | 14.18 | 6.10 | 4.08 | 9.12 |
| Dates and times of the ambulance calls | | | |  | | |
| Weekday daytime (9–16) | Ref | | | Ref | | |
| Weekday early night (17–0) | 1.14 | 0.92 | 1.43 | 1.17 | 0.92 | 1.48 |
| Weekday late night (1–8) | 1.05 | 0.81 | 1.35 | 1.11 | 0.83 | 1.49 |
| Weekend daytime (9–16) | 0.86 | 0.64 | 1.14 | 0.78 | 0.46 | 1.33 |
| Weekend early night (17–0) | 0.85 | 0.63 | 1.15 | 0.78 | 0.61 | 0.99 |
| Weekend late night (1–8) | 1.34 | 0.96 | 1.87 | 1.35 | 0.97 | 1.88 |
| Number of hospital inquiries | | | |  |  |  |
| <4 | Ref | | | Ref | | |
| ≥4 | 61.51 | 40.07 | 94.43 | 71.23 | 48.00 | 105.70 |
| *The pre-pandemic period was between January 2016 and March 2020.  † To adjust for possible geographical variations, the fire stations from which the ambulances were dispatched were included as dummy variables.  The categories of newborns, water-related accidents, and others were not shown because they did not have an outcome of prolonged OST. | | | | | | |

COVID-19, Coronavirus disease 2019; OST, on-scene time; OR, odds ratio; CI, confidence interval
