# Supplementary material for: Factors associated with prolonged on-scene time in ambulance transportation among patients with minor diseases or injuries in Japan: a population-based observational study
Source: BMC Emerg Med. 2024 Jan 7;24:10. doi: 10.1186/s12873-023-00927-2 (PMC10773094; doi:10.1186/s12873-023-00927-2)
Supplement: Supplementary file 3 — Supplementary Material 3 [file 12873_2023_927_MOESM3_ESM.docx]

**Additional file 3**

Table S3. Characteristics of the fire stations

|  | Total participants  N (%)  (N=20,069) | Patients with  prolonged OST  (n=1,241, 6.2%)  n (% for N) |
| --- | --- | --- |
| Fire station |  |  |
| Higashihiroshima Fire Station | 8,216 (40.9) | 554, 6.7% |
| Higashihiroshima Fire Station North Substation | 3,402 (17.0) | 182, 5.3% |
| Higashihiroshima Fire Station South Substation | 2,203 (11.0) | 115, 5.2% |
| Higashihiroshima Fire Station East Substation | 1,603 (8.0) | 83, 5.2% |
| Higashihiroshima Fire Station West Substation | 895 (4.5) | 35, 3.9% |
| Higashihiroshima Fire Station Akitsu Substation | 880 (4.4) | 81, 9.2% |
| Takehara Fire Station | 2,127 (10.6) | 142, 6.7% |
| Takehara Fire Station Tadanoumi Substation | 454 (2.3) | 38, 8.4% |
| Osakikamijima Fire Station | 289 (1.4) | 11, 3.8% |

OST, on-scene time

| Table S4. Odds ratios (ORs) and 95% confidence intervals (CIs) for prolonged on-scene time (OST) that excluded participants transported by ambulance from the Osakikamijima Fire Station (n=19,760): Results of univariable and multivariable logistic regression analyses (Analysis S3) | | | | | | |
| --- | --- | --- | --- | --- | --- | --- |
|  | Crude | | | Adjusted^*^ | | |
|  | OR | 95% CI | | OR | 95% CI | |
| Age |  | | |  | | |
| Infants | 0.47 | 0.35 | 0.62 | 0.53 | 0.39 | 0.71 |
| Adolescents | 0.74 | 0.56 | 0.99 | 0.80 | 0.54 | 1.19 |
| Adults | Ref | | | Ref | | |
| Older people | 0.98 | 0.87 | 1.11 | 1.19 | 1.03 | 1.38 |
| Sex |  | | |  | | |
| Male | Ref | | | Ref | | |
| Female | 1.06 | 0.94 | 1.19 | 1.13 | 1.08 | 1.18 |
| Accident type |  | | |  | | |
| Acute illnesses | Ref | | | Ref | | |
| Fire accidents | 5.86 | 3.23 | 10.61 | 7.78 | 3.81 | 15.91 |
| Natural disasters | 16.40 | 5.74 | 46.89 | 28.90 | 2.04 | 410.35 |
| Motor vehicle accidents | 1.37 | 1.19 | 1.59 | 1.63 | 1.29 | 2.06 |
| Work-related accidents | 1.14 | 0.63 | 2.05 | 1.54 | 0.78 | 3.04 |
| Sport-related accidents | 0.23 | 0.06 | 0.92 | 0.20 | 0.04 | 0.97 |
| Other types of accidents | 0.92 | 0.78 | 1.09 | 0.92 | 0.79 | 1.08 |
| Assaults | 2.77 | 1.53 | 5.01 | 2.97 | 1.94 | 4.55 |
| Self-injuries | 6.56 | 4.06 | 10.60 | 5.67 | 3.37 | 9.52 |
| Others | 2.73 | 0.33 | 22.74 | 3.13 | 0.25 | 39.11 |
| Dates and times of the ambulance calls | | | |  | | |
| Weekday daytime (9–16) | Ref | | | Ref | | |
| Weekday early night (17–0) | 0.98 | 0.84 | 1.14 | 0.99 | 0.81 | 1.22 |
| Weekday late night (1–8) | 0.94 | 0.79 | 1.12 | 0.95 | 0.63 | 1.44 |
| Weekend daytime (9–16) | 0.71 | 0.57 | 0.87 | 0.69 | 0.43 | 1.09 |
| Weekend early night (17–0) | 0.68 | 0.54 | 0.85 | 0.66 | 0.53 | 0.83 |
| Weekend late night (1–8) | 1.00 | 0.78 | 1.28 | 0.99 | 0.78 | 1.25 |
| Number of hospital inquiries | | | |  |  |  |
| <4 | Ref | | | Ref | | |
| ≥4 | 68.09 | 48.43 | 95.72 | 77.71 | 53.76 | 112.35 |
| COVID-19 pandemic period | | | |  |  |  |
| Pre-pandemic period  (1/2016–-3/2020) | Ref  Ref | | | Ref | | |
| Pandemic period  (4/2020–-12/2020) | 1.91 | 1.70 | 2.14 | 2.02 | 1.62 | 2.51 |
| *To adjust for possible geographical variations, the fire stations from which the ambulances were dispatched were included as dummy variables.  The categories of newborns and water-related accidents were not shown because they did not have an outcome of prolonged OST. | | | | | | |

COVID-19, Coronavirus disease 2019; OST, on-scene time; OR, odds ratio; CI, confidence interval
